# Intimate partner violence during pregnancy in COVID-19 pandemic: a cross-sectional study from South-west of Iran

**DOI:** 10.1186/s12889-023-15258-x

**Published:** 2023-02-14

**Authors:** Najmeh Maharlouei, Shohreh Roozmeh, Mohammad-hassan Zahed Roozegar, Hadi Raeisi Shahraki, Khadijeh Bazrafshan, Shaghayegh Moradi-alamdarloo, Hossein Molavi Vardanjani, Kamran B. Lankarani

**Affiliations:** 1 Health Policy Research Center, Institute of Health, Shiraz University of Medical Sciences, Shiraz, Iran; 2 Maternal-Fetal Medicine Research Center, Shiraz University of Medical Sciences, Shiraz, Iran; 3 Department of Epidemiology and Biostatistics, Faculty of Health, Shahrekord University of Medical Sciences, Rahmatieh Educational Complex, Shahrekord, Iran; 4 Department of MPH, Shiraz Medical School, Shiraz University of Medical Sciences, Shiraz, Iran

**Keywords:** Intimate partner violence, Pregnancy, Abuse

## Abstract

**Background:**

Intimate partner violence (IPV) against pregnant women can cause several complications for the mother and her baby, which are life-threatening. Thus, we aimed to find the prevalence of IPV and its associated factors in pregnant women in Shiraz, Iran.

**Methods:**

This cross-sectional study was conducted among pregnant mothers in Shiraz between July 2020 and January 2021. The questionnaire consisted of four parts: demographic data, socio-economic status (SES), obstetric and medical history, and questions about IPV. Univariate analysis was performed using Chi-square, McNemar, or Fisher’s exact test, and variables with p-value < 0.20 were included in Logistic regression. The odds ratio and CI 95% for variables with p-value < 0.05 were considered statistically significant.

**Results:**

The overall prevalence of IPV was 93.1% among 830 pregnant women in Shiraz. Psychological violence was the most prevalent type (92.9%), followed by sexual (11%) and physical (7.7%) violence. High SES (OR = 3.21, (CI:1.61–6.41)) was the only risk factor for overall violence, and the age group, 30–34, was a risk factor for physical violence. Mother-desired pregnancy (OR = 26 (Cl:0.09–0.79)) and father-desired pregnancy (OR = 0.91, (CI:0.22–3.80)) were protective factors against physical and sexual violence, respectively. Furthermore, Psychological violence and sexual violence increased during COVID-19 Pandemic (P.value < 0.05).

**Conclusion:**

According to the obtained results, the prevalence of IPV during pregnancy in Shiraz was very concerning, especially psychological violence. Improving conflict-solving skills among family members and addressing economic problems could be considered by health policymakers when designing interventional programs and policies to reduce IPV during pregnancy.

## Background

World Health Organization (WHO) defines intimate partner violence as any behavior within an intimate relationship that causes physical, sexual, or psychological harm, including physical aggression, sexual coercion, psychological abuse, and/or controlling behaviors by current or former spouses or partners [1]. Globally, WHO estimates that 30% of women experience IPV during their lifetime [1]. IPV increases the risk of stress-related, medical, and gynecological issues in women, including depression, substance use, cardiovascular disease, and poor reproduction [2, 3]. Further, IPV increases the risk of sexually transmitted diseases (STDs) [4].

IPV during pregnancy will affect the mothers’ health during and after pregnancy, including miscarriage, preterm labor, and postpartum depression [2, 5–9]. It also affects the newborn well-being through low birth weight, developmental problems, and behavioral misconduct in the future [2, 5–9]. In addition, IPV imposes a remarkably high-cost burden on healthcare systems [10].

According to the WHO report, Iran’s IPV rate was more than 30% [1]. Also, in 2018, the lifetime prevalence of overall IPV was reported to be more than 50% in Shiraz, the 5th most populous city in Iran [11]. Furthermore, some studies showed an increased rate of IPV during the Coronavirus 2019 (COVID-19) pandemic worldwide [12, 13]. The associated factors to the increased rate were low SES, unemployment, having toddlers, overcrowding families, and lockdowns by accentuating family stress levels [12–14]. Notably, pregnant women were susceptible to experiencing IPV [12, 13].

Like many other countries, Iran experienced lockdown and remote working during the COVID-19 pandemic [15–17]. Schools and universities became online, potentially increasing contact and family stress, as shown in other countries [12, 18]. The economic crisis also worsened during the COVID-19 pandemic, which could lead to an increased risk of IPV [11–13, 19, 20]. A population-based online study in Iran showed a significant increase in IPV (37.5%) during the COVID-19 pandemic and lockdowns [14].

Considering the impact of the COVID-19 pandemic on the IPV rate and the lack of data about IPV during pregnancy in recent years in Shiraz, Iran, we decided to conduct this Study to find the prevalence of IPV and its associated factors in pregnant women.

## Methods

### Setting and participants

This cross-sectional study was conducted on pregnant women from July 2020 to January 2021 in Shiraz, a city in South-west Iran and the capital of Fars province. The population of Shiraz is estimated to be 2,200,000 inhabitants, whose ethnicity consists of Fars, Lur, Turk, Arab, and Balooch, each with its unique culture.

The sample size was calculated based on the Study of Sobhani and co-workers [8]. Applying the prevalence of IPV (48%), the marginal error of 5%, and the error type one (0.05), the sample size was estimated as 383, and by considering the effect size of 2, the final sample size was considered 766 participants.

For choosing participants among pregnant women, we used maternity clinics located in Shiraz. We classified the maternity clinics into teaching, governmental, and private clinics. We did this categorization to estimate the participants’ socio-economic status (SES), as maternity care was the cheapest in prenatal teaching clinics affiliated with the teaching obstetric hospitals, while it was the most expensive in private clinics. The clinics were chosen randomly; then, based on the proportion of each clinic’s patients, we took samples through a mixture of randomized and convenient sampling. We chose the first three days of the even weeks each month for data collection and continued that to reach the calculated sample size.

Every pregnant Iranian woman who had lived in Shiraz for at least six months before the Study and was interested in participating in this study was chosen. We excluded those pregnant mothers who came to the clinic with a companion to reduce the information bias. Considering the sensitive concept of this study, we trained the secretary of the clinics to explain the idea and the aim of the Study to pregnant mothers who would refer to that clinic for maternity care. After obtaining the informed consent form of all participants, they were asked to answer an anonymous questionnaire, either the online format using a tablet lent to them or a printed version of the questionnaire based on the mother’s convenience. A private place was set up for answering the questionnaire. Also, the participants were instructed to fill out the questionnaire, and they could ask questions about the clinic’s secretary’s concept (if any). Furthermore, the pregnant mother was asked to put their contact information if they needed any help from investigators of this project.

### Data collection

Since no standard questionnaire had been made in Persian for IPV assessment in pregnant women, we used a questionnaire used in a study in Ethiopia [21]. We used a questionnaire for gathering data, which consisted of four parts; demographic data, SES, obstetric and medical history, and IPV. After using the backward-forward translation method, the preliminary format of the questionnaire was sent to an expert panel consisting of an epidemiologist, a midwife, two gynecologists, and a community medicine specialist to check its validity. Also, we assessed one hundred pregnant women in a pilot study to check the reliability of the questionnaire, which showed a consistency of over 80%. The participants in the pilot study were later included as participants in the Study.

The violence part of this questionnaire had three sub-sections: physical violence (six questions), psychological violence consisting of eight questions on controlling the behavior of a spouse, and five questions on emotional violence and sexual violence (three questions). At the beginning of each sub-section, we wrote a sentence to explain the concept and gave some examples for more clarity. Each question had four Likert-scale choices: Never, Sometimes, Usually, and Always/Every day. Then, “Never” was recoded to “zero”, and all other choices were recoded to “one”. Participants were asked whether the frequency of each type of violence had changed during the COVID-19 pandemic and pregnancy. The respondents were instructed to choose one of the following choices: “not changed”, “has been decreased”, and “has been increased”.

### Statistical analysis

Statistical analysis was performed in the SPSS software version 25, and P < 0.05 was considered statistically significant. Descriptive statistics were reported as mean (± SD) or frequency (%), and univariate analysis was performed using Chi-square, McNemar, or Fisher’s exact test. Finally, variables with P-value < 0.20 in the univariate analysis were included in the logistic regression models to investigate adjusted odds ratios.

### Ethics

The study protocol was written based on the Helsinki ethical principles for medical research and approved by the Ethics Committee affiliated with Shiraz University of Medical Sciences (SUMS) (IR.SUMS.REC.1400.387). The questionnaire did not contain any information about the name or phone numbers of the participants to respect their privacy. However, participants were offered free psychological counseling if they thought they needed it. Also, informed consent was obtained from all participants.

## Results

Of 830 pregnant women, 315 (38.0%) were 30 to 34 years old, and 344 (41.4%) had a high school diploma. The marriage duration of 319 (38.4%) participants was 5 to 10 years, and almost all (99.5%) lived with their husbands. Furthermore, most of our participants were housewives (91.6%), reported the SES of their family as middle class (42.7%), had Fars ethnicity (70.2%), and resided in the urban area (75.7%).

**Fig. 1:**
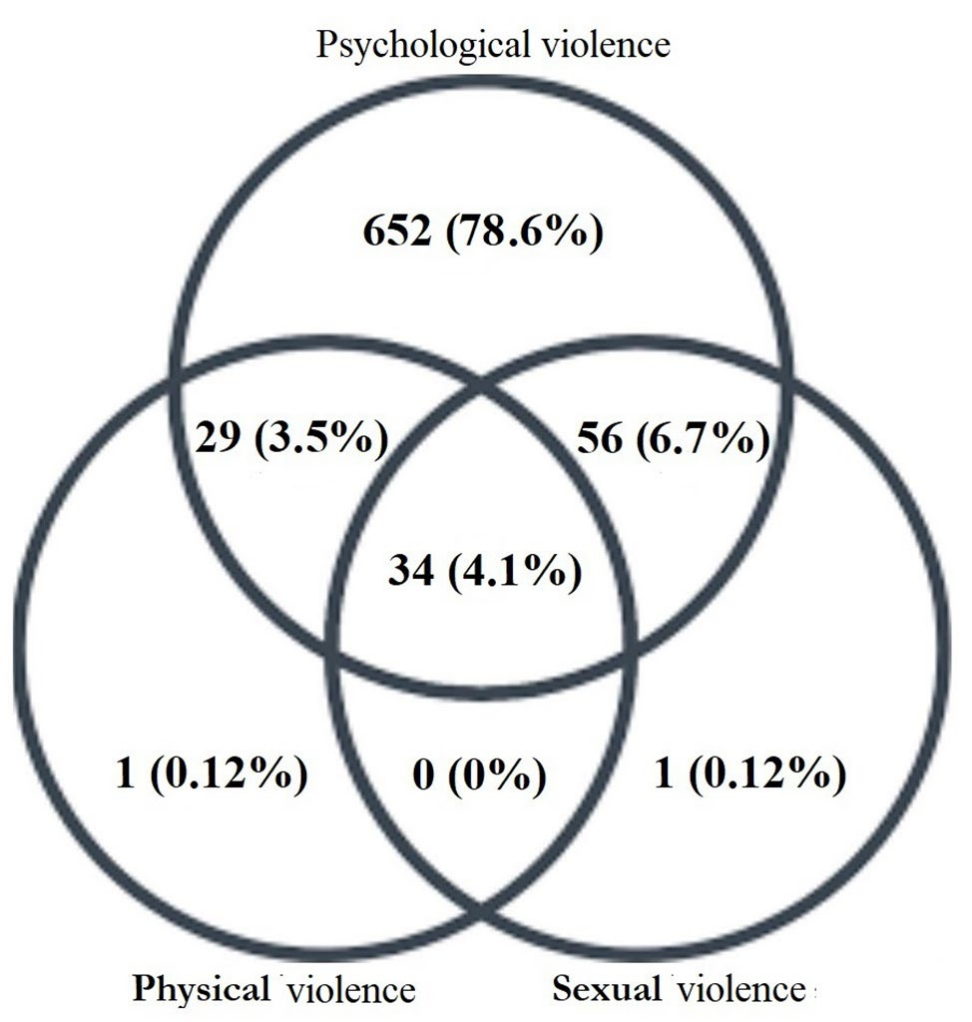
Frequency of violence in pregnant women

We found that 773 pregnant mothers (93.1%) reported at least one type of violence during this pregnancy, considered the overall violence, and 771 (92.9%) had experienced psychological violence. In addition, 64 (7.7%) and 91 (11.0%) pregnant mothers reported physical and sexual violence, respectively (Fig. 1).

The frequency of reported sexual violence was significantly lower among pregnant women with a university degree (6.4%) compared to expecting mothers who had a high school diploma (13.1%) or those who did not finish high school (12.3%). Although the frequency of overall violence was significantly lower in pregnant women with low SES (88.7%) compared to those with middle (96.6%) or high (93.7%) SES, the frequency of physical and sexual violence was significantly lower in the high socio-economic class (1.7% and 2.9%) compared to their counterparts from low SES (4.3% and 7.6%) and middle SES (13.6% and 17.8%). Moreover, mothers whose pregnancy was planned experienced a lower frequency of physical (7.0% versus 25.0%, P = 0.002) and sexual violence (10.2% versus 31.3%, P = 0.001). Still, a higher rate of psychological violence (93.4% versus 81.3%, P = 0.02) compared to the remainder of the participants (Table 1).

**Table 1:** Association between demographic variables and different types of violence

Variable	Frequency (%)		Overall violence	Physical violence	Psychological violence	Sexual violence
**Age (year)**						
< 25	134 (16.1)		122 (91.0)	5 (3.7)	122 (91.0)	14 (10.4)
25–29	171 (20.6)		159 (93.0)	13 (7.6)	159 (93.0)	12 (7.0)
30–34	315 (38.0)		291 (92.4)	34 (10.8)	289 (91.7)	41 (13.0)
35–39	161 (19.4)		155 (96.3)	10 (6.2)	155 (96.3)	21 (13.0)
> 39	49 (5.9)		46 (93.9)	2 (4.1)	46 (93.9)	3 (6.1)
**P-value**	-		0.45	0.07	0.38	0.20
**Education status**						
Under diploma	252 (30.4)		229 (90.9)	21 (8.3)	229 (90.9)	31 (12.3)
Diploma	344 (41.4)		320 (93.0)	29 (8.4)	320 (93.0)	45 (13.1)
University degree	234 (28.2)		224 (95.7)	14 (6.0)	222 (94.9)	15 (6.4)
**P-value**	-		0.11	0.50	0.23	0.03*
**Job**						
Housewife	760 (91.6)		706 (92.9)	62 (8.2)	704 (92.6)	88 (11.6)
Employed	70 (8.4)		67 (95.7)	2 (2.9)	67 (95.7)	3 (4.3)
P-value	-		0.47	0.11	0.47	0.06
**Parents living in the same house**						
Yes	826 (99.5)		770 (93.2)	64 (7.7)	768 (93.0)	91 (11.0)
No	4 (0.5)		3 (75.0)	0 (0)	3 (75.0)	0 (0)
**P-value**	-		0.25	0.99	0.26	0.99
**Socio-economic status**						
Low	302 (36.4)		268 (88.7)	13 (4.3)	267 (88.4)	23 (7.6)
Middle	354 (42.7)		342 (96.6)	48 (13.6)	341 (96.3)	63 (17.8)
High	174 (21.0)		163 (93.7)	3 (1.7)	163 (93.7)	5 (2.9)
**P-value**	-		< 0.001*	< 0.001*	< 0.001*	< 0.001*
**Residence area**						
City	628 (75.7)		585 (93.2)	48 (7.6)	583 (92.8)	62 (9.9)
Rural	202 (24.3)		188 (93.1)	16 (7.9)	188 (93.1)	29 (14.4)
P-value	-		0.97	0.90	0.91	0.08
**Ethnicity**						
Fars	583 (70.2)		545 (93.5)	37 (6.3)	543 (93.1)	60 (10.3)
Lor	117 (14.1)		107 (91.5)	13 (11.1)	107 (91.5)	12 (10.3)
Tork	99 (11.9)		90 (90.9)	11 (11.1)	90 (90.9)	14 (4.1)
Other	31 (3.7)		31 (100)	3 (9.7)	31 (100)	5 (16.1)
**P-value**	-		0.30	0.16	0.34	0.54
**Marriage duration (years)**						
< 5	272 (32.8)		256 (94.1)	19 (7.0)	256 (94.1)	25 (9.2)
5–10	319 (38.4)		297 (93.1)	22 (6.9)	296 (92.8)	33 (10.3)
> 10	239 (28.8)		220 (92.1)	23 (9.6)	219 (91.6)	33 (13.8)
**P-value**	-		0.65	0.42	0.55	0.23
**Number of living children**						
1	283 (34.1)		264 (93.3)	17 (6.0)	264 (93.3)	28 (9.9)
2	279 (33.6)		259 (92.8)	21 (7.5)	258 (92.5)	28 (10.0)
3	164 (19.8)		156 (95.1)	16 (9.8)	156 (95.1)	21 (12.8)
>= 4	104 (12.5)		94 (90.4)	10 (9.6)	93 (89.4)	14 (13.5)
**P-value**	-		0.51	0.45	0.35	0.61
**Mother wanted the baby**						
No	32 (3.9)		26 (81.3)	8 (25.0)	26 (81.3)	10 (31.3)
Yes	798 (96.1)		747 (93.6)	56 (7.0)	745 (93.4)	81 (10.2)
**P-value**	-		0.02*	0.002*	0.02*	0.001*
**Father wanted the baby**						
No	27 (3.3)		23 (85.2)	8 (29.6)	23 (85.2)	11 (40.7)
Yes	803 (96.7)		750 (93.4)	56 (7.0)	748 (93.2)	80 (10.0)
**P-value**	-		0.11	0.001*	0.12	< 0.001*
**Sex of the fetus**						
Boy	328 (39.5)		307 (93.6)	26 (7.9)	306 (93.3)	40 (12.2)
Girl	340 (41.0)		313 (92.1)	29 (8.5)	312 (91.8)	38 (11.2)
Unknown at the time of the interview	162 (19.5)		153 (94.4)	9 (5.6)	153 (94.4)	13 (8.0)
**P-value**	-		0.56	0.50	0.52	0.38
**Spacing**						
< 3 years	463 (55.8)		432 (93.3)	36 (7.8)	431 (93.1)	49 (10.6)
>= 3 years	367 (44.2)		341 (92.9)	28 (7.6)	340 (92.6)	42 (11.4)
**P-value**	-		0.89	0.99	0.89	0.74

Spacing: The duration between the last two pregnancies (if applicable)

*A P-value less than 0.05 was considered significant

The logistic regression analysis revealed that mothers aged 30 to 34 had a higher experience of physical violence than their counterparts younger than 25 (OR = 3.42, 95%CI: 1.27–9.22). In addition, compared to low SES participants, those from high SES reported significantly higher physical violence (OR = 4.53, 95%CI: 2.27–9.06), psychological violence (OR = 3.34, 95%CI: 1.72–6.47), sexual violence (OR = 3.45, 95% CI 2.00-5.96), and the overall violence (OR = 3.21, 95%CI: 1.61–6.41) (Table 2). Furthermore, maternal desire toward having a baby was a protective factor against physical violence (OR = 0.26, 95% CI: 0.09–0.79), and desiring pregnancy from the father’s side was a protective factor for experiencing sexual insult during pregnancy (OR = 0.20, 95% CI 0.07–0.56) (Table 2).

**Table 2:** Results of logistic regression models for the association of variables with different types of violence

Variable	Overall violence OR (CI 95%)	Physical violence OR (CI 95%)	Psychological violence OR (CI 95%)	Sexual violence OR (CI 95%)
**Age (year)**				
< 25		Baseline		
25–29		1.87 (0.63–5.53)		
30–34		3.42 (1.27–9.22)*		
35–39		1.85 (0.60–5.75)		
40 and higher		1.21 (0.20–7.25)		
**Education status**				
Less than diploma	Baseline			Baseline
High school diploma	1.22 (0.66–2.24)			1.21 (0.72–2.04)
University degree	1.85 (0.80–4.30)			0.66 (0.32–1.33)
**Job**				
Housewife		Baseline		Baseline
Employed		0.30 (0.06–1.48)		0.42 (0.12–1.49)
**Socio-economic status**				
Low	Baseline	Baseline	Baseline	Baseline
Moderate	1.42 (0.65–3.09)	0.53 (0.14–1.97)	1.86 (0.91–3.79)	0.60 (0.22–1.69)
High	3.21 (1.61–6.41)*	4.53 (2.27–9.06)*	3.34 (1.72–6.47)*	3.45 (2.00-5.96)*
**Residential area**				
City				Baseline
Rural				1.40 (0.84–2.33)
**Ethnicity**				
Fars		Baseline		
Lor		1.76 (0.85–3.64)		
Tork		1.57 (0.74–3.34)		
Other		1.55 (0.41–5.90)		
**Mother wanted pregnancy**				
No	Baseline	Baseline	Baseline	Baseline
Yes	3.14 (0.95–10.36)	0.26 (0.09–0.79)*	2.93 (0.89–9.61)	0.40 (0.15–1.11)
**Father wanted pregnancy**				
No	Baseline	Baseline	Baseline	Baseline
Yes	0.91 (0.22–3.80)	0.33 (0.10–1.03)	0.95 (0.23–3.95)	0.20 (0.07–0.56)*

*A P-value less than 0.05 was considered significant

**Fig. 2:**
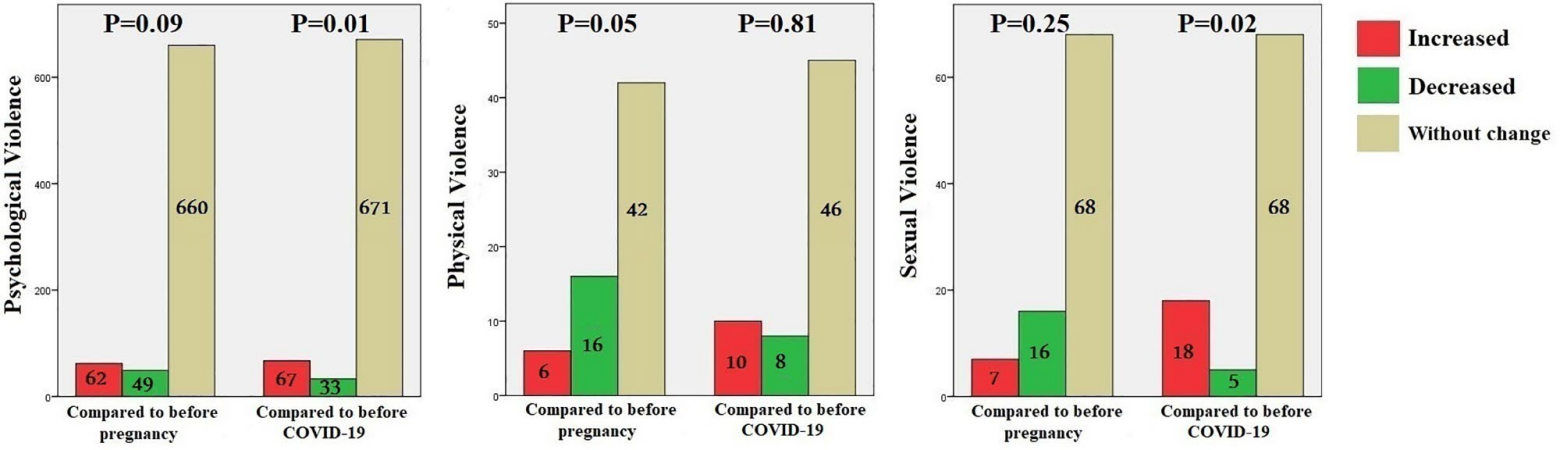
Violence occurrence compared to before pregnancy and before the COVID-19 pandemic by different types of violence

Violence was compared between pregnancy and pre-pregnancy as well as between the COVID-19 pandemic and before that era. The analysis demonstrated that none of the violence sub-types had significantly changed compared to pre-pregnancy. However, psychological violence (p-value = 0.01) and sexual violence (p-value = 0.02) increased during the COVID-19 pandemic compared to before that era. (Fig. 2)

## Discussion

According to our findings, nine out of ten pregnant women had experienced at least one type of violence during their current pregnancy. Also, this study revealed that all sorts of IPV, including physical, psychological, and sexual, were reported more frequently in participants with high SES. In contrast, the desire to have a baby was a protective factor in all types of IPV; however, based on statistical modeling, fathers’ desire for the baby was a protective factor against sexual violence, and mothers’ desire for the baby was a protective factor against physical violence. We also found that IPV frequency had no significant change during pregnancy, while psychological violence and sexual violence increased during the COVID-19 pandemic compared to before that era.

According to our study, the prevalence of violence against pregnant women was 93.1%, much higher than overall violence against non-pregnant women in Shiraz in 2018, which was reported at 50% [11]. It was also approximately three times higher than the IPV reported by pregnant mothers from other developing countries such as Pakistan and India [21, 22]. There are three possible reasons for this remarkable increase compared to the previous studies in Shiraz and other developing countries in Asia. First, our study might provide more comprehensive definitions of different types of violence on the questionnaire so that the respondents could answer the questions more precisely and reliably. Secondly, the new generation of women may be braver in reporting violence without fear of stigmatization, so the rate of IPV has increased. Thirdly, our Study was conducted during the recent pandemic, when the level of IPV increased [12–14].

Furthermore, our Study indicated that the high overall violence rate during pregnancy was primarily due to the high prevalence of psychological violence. This finding was also similar to the Study of Rurangirwa et al., who reported the psychological type of IPV as the highest prevalent type of violence during pregnancy [23]. The higher rate of psychological violence compared to other types may be rooted in fear of harming the unborn baby by committing physically violent behavior [24].

Previous studies demonstrated that SES was a protective factor against violence in pregnant and non-pregnant women [5, 26, 28]. Our research revealed that higher SES was a risk factor for exposure to physical, psychological, and sexual violence. The reason might be that participants with high SES were more frank and brave in reporting different types of violence, showing a change in women’s beliefs and behaviors compared to the past [25].

Moreover, we found physical violence more prevalent in high SES families compared to their counterparts belonging to lower SES, which was inconsistent with the previous studies [5, 29]. Therefore, SES should be cautiously considered as a risk factor for physical violence as it could play different roles based on the culture of the studied community. Additionally, logistic regression modeling showed that pregnant women in the age group of 30–34 had a higher risk of exposure to physical violence by their spouse, which was confirmed by previous studies [26, 28].

In this study, psychological violence increased with SES, which was inconsistent with the results of the studies conducted by Rurangiwa et al. and Moazen et al. [11, 23]. This controversy could be due to more financial independence of higher SES pregnant mothers, which made them more courageous in reporting violence and less fearful of facing the consequences.

We found the desire for the baby as a protective factor against IPV. The mother-desired pregnancy was a protective factor against experiencing physical violence, and the father-desired pregnancy was a protective factor against sexual violence. These two findings were similar to Karaoglu et al.’s study [24], probably due to fear of the stigma of hurting pregnant women through physically or sexually violent behavior [24].

Moreover, we found most respondents were violated psychologically and sexually at a higher level than before the pandemic. Iran has been struggling with an economic crisis caused by international sanctions [19], worsened during the COVID-19 pandemic [20]. Studies have shown that the growth of economic issues has played an essential role in increasing the rate of IPV, especially during the COVID-19 pandemic. [11–13]. Other possible factors could be more hours the household had to spend with each other at home due to the lockdown, remote working, and online education of the students during the COVID-19 pandemic [12]. Hence, providing families with family conflict resolution skills could be highly beneficial to prevent IPV.

### Limitations, strengths, and recommendations

Regarding limitations, we should mention that this Study was cross-sectional, in which we asked the participants to report the changes in IPV during pregnancy and the COVID-19 pandemic. However, asking the participants to fill in the questionnaires at preconception visits and then at a visit in the third trimester could present more reliable results. Moreover, we executed the Study in Shiraz, the fifth most populous city in Iran; hence, the results might not be reproducible for regions with various proportions of educated pregnant mothers and different cultures. Therefore, a more comprehensive survey at the national level is recommended. Nevertheless, this study is unique in estimating the prevalence of IPV in expectant mothers during the COVID-19 pandemic in Shiraz, Iran.

## Conclusion

The high prevalence of IPV during pregnancy in Shiraz is very concerning especially psychological violence, which needs immediate interventions. To minimize the IPV rate in pregnant mothers, especially during situations that increase tensions in families applying appropriate policies by health policymakers and stakeholders should be considered. Some practical strategies can be improving conflict-solving skills through educational programs to empower families and address the financial issues of the citizens.

## Data Availability

Since some participants applied to be visited by a psychiatrist and their personal information, including their name and contact number, was registered (not in the questionnaire), it is impossible to be publicized. So, the datasets used and analyzed during the Study are available from the corresponding author upon reasonable request.

## List of abbreviations

IPV: Intimate partner violence
WHO: World Health Organization
SES: Socio-economic status
SUMS: Shiraz University of Medical Sciences
